# Discovery of novel therapeutic properties of drugs from transcriptional responses based on multi-label classification

**DOI:** 10.1038/s41598-017-07705-8

**Published:** 2017-08-02

**Authors:** Lingwei Xie, Song He, Yuqi Wen, Xiaochen Bo, Zhongnan Zhang

**Affiliations:** 1 Software School, Xiamen University, Xiamen Fujian, 361005 P.R. China; 2 Beijing Institute of Radiation Medicine, Beijing, 100850 P.R. China

## Abstract

Drug repositioning strategies have improved substantially in recent years. At present, two advances are poised to facilitate new strategies. First, the LINCS project can provide rich transcriptome data that reflect the responses of cells upon exposure to various drugs. Second, machine learning algorithms have been applied successfully in biomedical research. In this paper, we developed a systematic method to discover novel indications for existing drugs by approaching drug repositioning as a multi-label classification task and used a Softmax regression model to predict previously unrecognized therapeutic properties of drugs based on LINCS transcriptome data. This approach to complete the said task has not been achieved in previous studies. By performing in silico comparison, we demonstrated that the proposed Softmax method showed markedly superior performance over those of other methods. Once fully trained, the method showed a training accuracy exceeding 80% and a validation accuracy of approximately 70%. We generated a highly credible set of 98 drugs with high potential to be repositioned for novel therapeutic purposes. Our case studies included zonisamide and brinzolamide, which were originally developed to treat indications of the nervous system and sensory organs, respectively. Both drugs were repurposed to the cardiovascular category.

## Introduction

Despite achieving considerable progress during the past several decades, traditional de novo research and development of drugs remains to be extremely costly, risky, and time consuming^1, 2^. In addition, several drugs already on the market have been repositioned for new therapeutic applications^3, 4^ because of their capacity to affect more than one molecular target with different pharmacological effects (also known as drug promiscuity). Moreover, multi-functions of drug targets have contributed to drug repurposing. This innovative paradigm in drug development is economically attractive, low risk, and time saving^5^. One successful example of drug repositioning is the expanded use of sildenafil, which was originally developed to treat angina but later repurposed for the treatment of erectile dysfunction and pulmonary hypertension^6, 7^.

Large-scale efforts to acquire biomedical data continue to generate extensive amounts of multi-omics data and pharmaceutical informatics data^8–10^. By utilizing transcriptome data, researchers can make systematic discoveries of new indications for approved drugs. The Connectivity Map (also known as CMap) database, launched in 2006, includes thousands of transcriptional profiles under various drug perturbations, thereby accelerating the speed of drug repositioning^11^. Based on the CMap database, Iorio F *et al*. constructed a drug similarity network and predicted that the approved drug fasudil could promote autophagy, and Liu J *et al*. predicted the new indication of celastrol for the treatment of obesity^12–14^.

In addition, machine learning algorithms with excellent performance in image, text, voice, robotics, bioinformatics, and autonomous driving have been used widely in biomedical research to generate numerous successful discoveries^15–21^. For instance, D’Ambrosio R *et al*. developed a reconstruction rule based on Softmax regression to solve multi-class classification tasks by using the error-correcting output code (ECOC) framework in various biological fields^22^. Sasaki Y *et al*. combined Softmax regression with a genetic algorithm for faster evolution speeds and evolvability control^23^. Combing support vector machines (SVM) with the *k*-nearest neighbor algorithm, Begum S *et al*. presented the SVM ensemble algorithm to identify the subtypes of cancer based on microarray data^24^.

In general, the application of machine learning for drug repositioning improves predictable and reliable pharmaceutical research and development. Napolitano F *et al*. proposed an approach to integrate multi-dimensional drug data and predicted the novel indications of drugs based on the structural similarity of drugs and drug–target relations^25^. However, the performance of machine learning algorithms in biomedical research fields remains a challenge because of data scarcity. Fusion of multi-omics data is beneficial for extending training data; however, noise is introduced. Therefore, we trained a multi-label classifier by gene-level transcriptomic data that could reflect internal attribution effectively^26^.

In 2010, the National Institute of Health (NIH) launched the Library of Integrated Network-based Cellular Signatures (LINCS) project, which aims to provide a comprehensive picture of multilevel cellular responses when cells are exposed to various perturbing agents (http://www.lincsproject.org/). The L1000 database of the LINCS project includes millions of genome-wide expression profiles gathered when 72 cell lines were stimulated by more than 20,000 small molecular compounds. This database provides the basis for the systematic discovery of drugs and facilitates the application of machine learning algorithms to drug repositioning.

In this paper, using a machine learning algorithm for multi-label classification, we systematically predicted new therapeutic properties of 480 approved drugs based on transcriptome data and under drug perturbations from the L1000 database. After a minimum of 200 iterations, our model learned how to provide a common representation of the original data from training sets with over 80% training accuracy, and the results exhibited 70% validation accuracy. We discovered that 98 drugs have high potential to be repositioned for novel therapeutic properties. Drugs with different therapeutic properties exhibit different repositioning potentials. When examining the data of drug side effects and structure, we found that if a drug is highly similar in these regards with other drugs that present certain therapeutic properties, the potential of the drug to be repositioned for the same therapeutic properties is improved as well. In a case study, we investigated zonisamide and brinzolamide, which are presently used for the treatment of selected indications in the nervous system and sensory organs, respectively, for possible repurposing to cardiovascular indications. Both drugs are similar to cardiovascular drugs in terms of structure and side effects.

## Results

### Machine learning results

**Figure 1:**
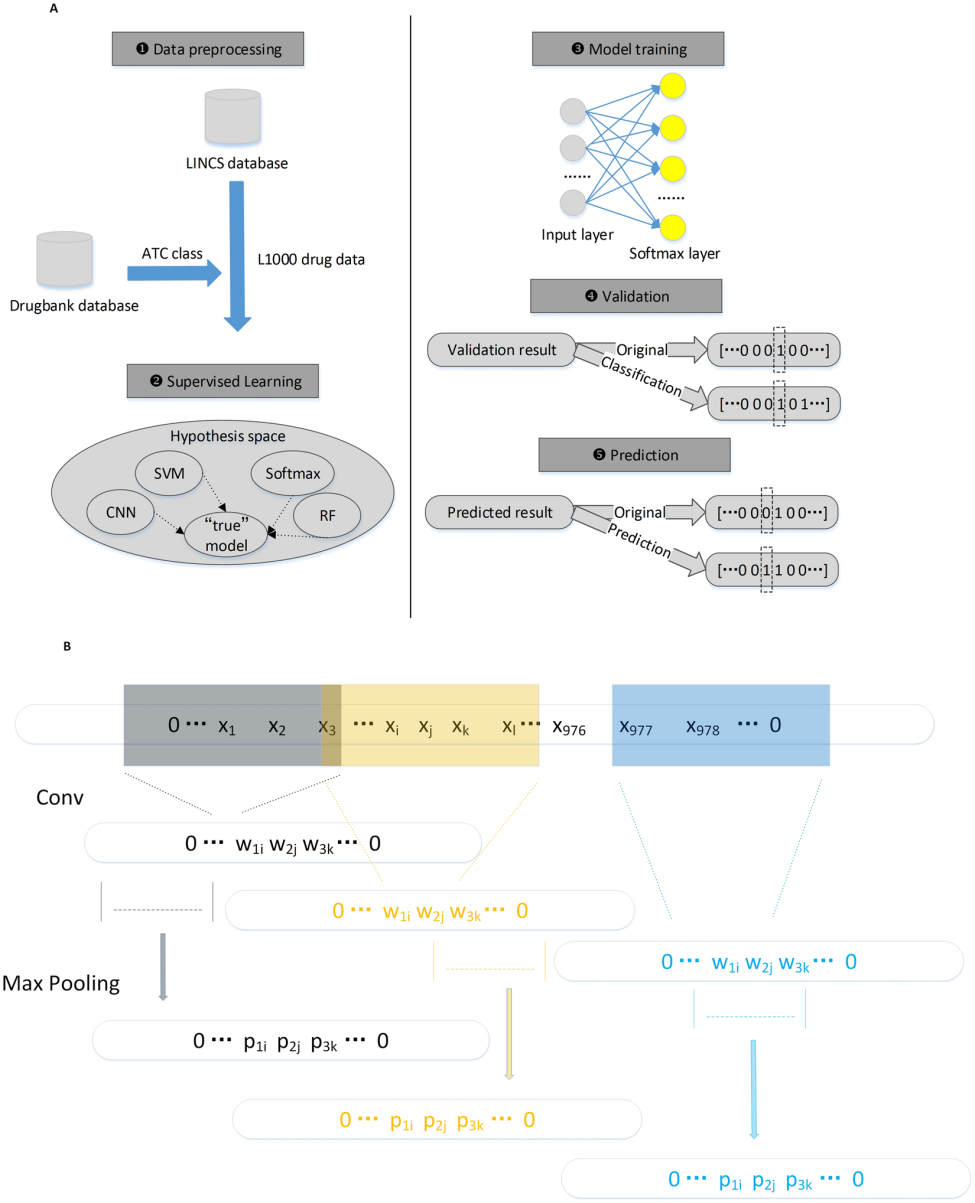
Total process and CNN architecture. (**A**) The whole process that contains data preprocess, supervised learning, model training, validation, and prediction. (**B**) The CNN architecture consists of two convolutional layers, two max pooling layers, one fully connected layer, and one classification layer. Different feature maps are extracted by various convolutional kernels based on sliding window and fed into max pooling layers for integrating local features.

In this work, drug repositioning was modeled as a multi-label classification task in the machine learning domain, as shown in Fig. 1A. Drug data containing 978 landmark genes and labels corresponding to Anatomical Therapeutic Chemical (ATC) classification were gathered from the LINCS and DrugBank databases. During supervised learning, we tried different generative models and discriminative models in the hypothesis space to fit the data. Multi-label SVM could be implemented efficiently with additional expensive computations, and the dataset was not linearly separable given that the number of support vectors (over 500) exceeded the number of classes. The performance of the random forest (RF) method, a common classifier that combines decision trees, depends on the integrity of feature values, but a small ratio of observed values to the full value range was obtained in this task^27–33^. Both SVM and RF encountered a challenge of class imbalance problems, especially for several classes with small proportions. Data synthesis is a common tool to avoid class imbalance, but no reliable approach can achieve this goal in this domain. In order to denoise and extract high-level features to represent raw data, we designed a convolutional neural network (CNN) that consists of convolution and max pooling operations (as shown in Fig. 1B). The convolution layers contained several kernels to extract different types of features. The max pooling layers are responsible for integrating local features to improve translation invariance. Even if different kernels in the same layer shared parameters, the structure of CNN is highly complicated to be fully trained. Moreover, we adopted libD3C that employed two types of selective ensemble techniques, which are a combination of the ensemble pruning based on *k*-means clustering and dynamic selection and circulating combination for comparison.

**Figure 2:**
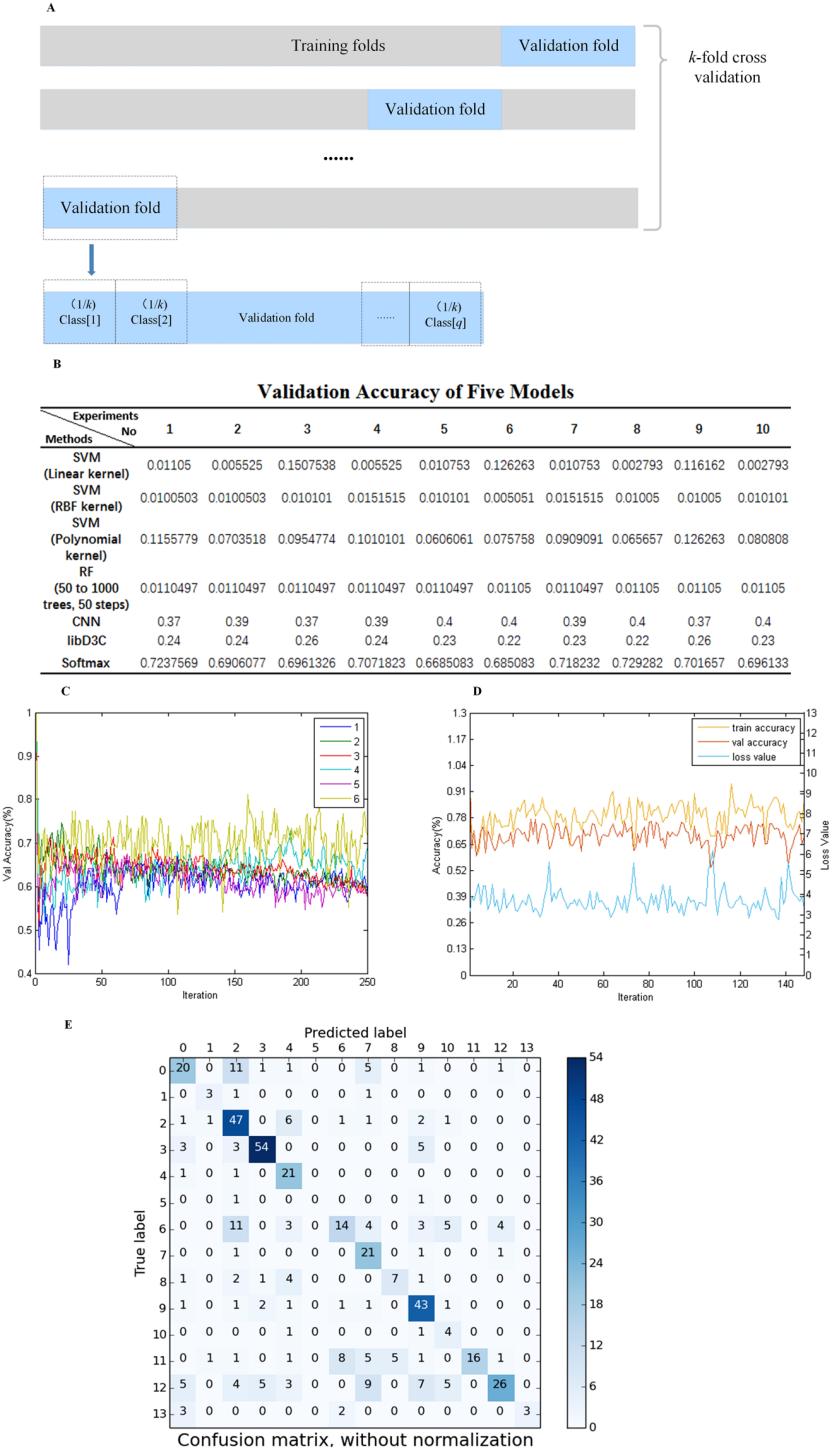
Experiment results of models. (**A**) *k*-fold cross-validation was used for setting hyper-parameters and evaluating generalizations. (**B**) The results from independent repeated experiments indicated that there was little prospect of prediction by using SVM with different kernel functions and RF. The mean performance of libD3C, which is the latest ensemble classifier, was slightly higher than 20%. The validation accuracy of CNN and Softmax was 40% versus 70%. (**C**) The learning curve of experimental results under different hyper-parameter settings, including learning rate, training threshold, validation threshold, and regularization weight. The best results were 0.06 of the learning rate, 0.30 of the training threshold, 0.06 of the validation threshold, and 1 of the regularization weight. (**D**) Softmax result under best hyper-parameter setting. The training accuracy was over 80%, and the validation accuracy was approximately 70%. (**E**) Confusion matrix representing Softmax classification performance. Each column of the matrix represents the instances in a predicted class, whereas each row represents the instances in an actual class.

Only Softmax, as a classifier, directly simplified the sophisticated structure and introduced inter-class competition for multi-label classification^34^. As shown in Fig. 2B, the performance of Softmax is markedly superior to those of the others based on results of repeated independent experiments. We used cross entropy as a cost function and mini-batch gradient descent algorithm to train the Softmax regression model with the best hyper-parameter setting, which was selected through observation of the learning curve on the *k*-fold cross-validation sets, as shown in Fig. 2A and C. The training process was GPU-accelerated. Finally, the new labels of new samples were predicted by the trained model. As shown in Fig. 2E, the false positive samples in the confusion matrix for a certain drug may indicate its potential for novel use or repurposing. This result indicates misclassification and may, therefore, lead to unexpected new discoveries.

The evaluation criteria for the classification results were defined as follows:

**Definition 1 True positive (TP):** For sample *i* and label *j*, *LB*_*i*,*j*_ is its given label, and *CF*_*i*,*j*_ is its predicted label. If *LB*_*i*,*j*_ = *CF*_*i*,*j*_ = 1, *CF*_*i*,*j*_ is a true positive prediction.

**Definition 2 True negative (TN):** For sample *i* and label *j*, *LB*_*i*,*j*_ is its given label, and *CF*_*i*,*j*_ is its predicted label. If *LB*_*i*,*j*_ = *CF*_*i,j*_ = 0, *CF*_*i*,*j*_ is a true negative prediction.

**Definition 3 False positive (FP):** For sample *i* and label *j*, *LB*_*i*,*j*_ is its given label, and *CF*_*i*,*j*_ is its predicted label. If *LB*_*i*,*j*_! = *CF*_*i*,*j*_ and *CF*_*i*,*j*_ = 1, *CF*_*i*,*j*_ is a false positive prediction.

**Definition 4 False negative (FN):** For sample *i* and label *j*, *LB*_*i*,*j*_ is its given label and *CF*_*i*,*j*_ is its predicted label. If *LB*_*i*,*j*_! = *CF*_*i*,*j*_ and *CF*_*i*,*j*_ = 0, *CF*_*i*,*j*_ is a false negative prediction.

**Definition 5 Correct sample prediction:** For sample *i*, if there exists *j* (1 ≤ *j* ≤ *q*), and *CF*_*i*,*j*_ is a true positive prediction, the prediction of sample *i* is correct, represented by *cp*(*i*) = 1.

When training accuracy converged, validation accuracy reached 70%, as shown in Fig. 2D. After repeated independent experiments, the mean accuracies of each ATC class were 73%, 40%, 84.87%, 66.54%, 62.5%, 25%, 68.57%, 65.38%, 52.08%, 82.04%, 72.5%, 50%, 63.84%, and 37.5%.

### Global analysis of novel therapeutic property

We averaged the results of the last 100 iterations and generated a probability matrix that indicated the potential of 480 drugs to be repositioned to 14 ATC therapeutic properties (Supplementary Table 1). Then, we set the probability of a drug being repositioned to its known ATC therapeutic property as 0. Thus, we constructed a drug therapeutic property network based on transcriptome data, denoted as DTN-T.

**Figure 3:**
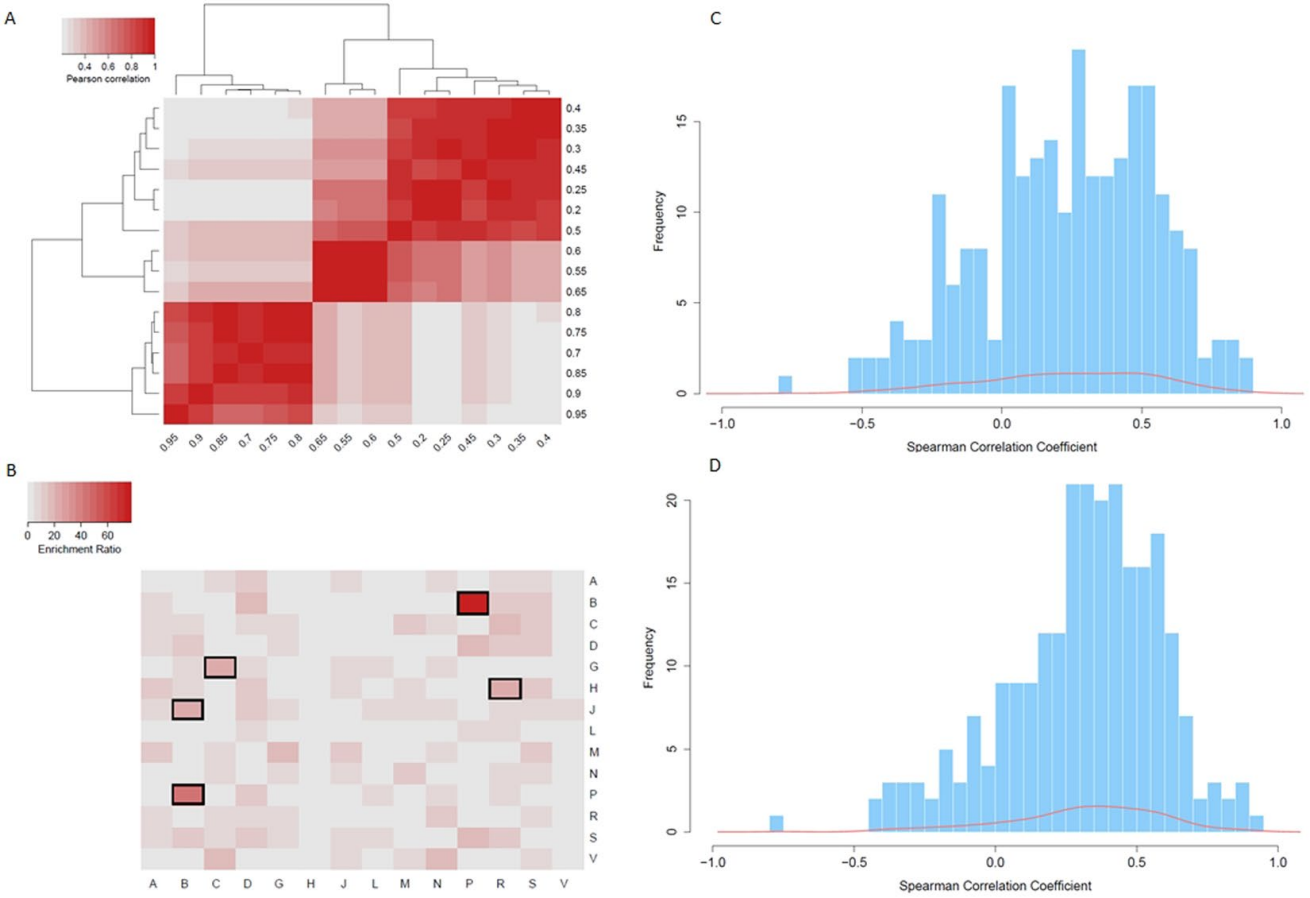
Global analysis of novel therapeutic property. (**A**) Pearson correlation coefficient of ER matrices with various DTN-T thresholds. (**B**) Heatmap of ER matrix indicating the repositioning potential between 14 therapeutic properties. ATC therapeutic properties include: A, alimentary tract and metabolism; B, blood and blood-forming organs; C, cardiovascular system; D, dermatologicals; G, genito-urinary system and sex hormones; H, systemic hormonal preparations, excluding sex hormones and insulins; J, anti-infectives for systemic use; L, anti-neoplastic and immunomodulating agents; M, musculoskeletal system; N, nervous system; P, anti-parasitic products; R, respiratory system; S, sensory organs; and V, several others. (**C**) Spearman correlation coefficient distribution of 247 drugs across DTN-T and DTN-SE. (**D**) Spearman correlation coefficient distribution of 247 drugs across DTN-T and DTN-ST.

To explore the repositioning potential among 14 therapeutic properties, we calculated the Enrichment Ratio matrix (ER matrix). A high ER of the therapeutic property X repositioned to therapeutic property Y indicated a high potential that therapeutic property X could be repositioned to the therapeutic property Y. We pruned the DTN-T according to thresholds from 0.2–0.95 and calculated the corresponding ER matrices. Next, we computed the Pearson correlation coefficient of the ER matrices with various DTN-T thresholds (Fig. 3A). The results demonstrated that the ER matrices with thresholds from 0.7–0.95 possess high correlation coefficients. Therefore, we combined and averaged the six ER matrices with the highest thresholds and highest correlation coefficients. The heatmap of combined ER matrices is illustrated in Fig. 3B. The top five ER values were B → P(ER = 77.8), P → B(ER = 42.4), J → B(ER = 23.1), H → R(ER = 22.2), and G → C(ER = 20.7), suggesting that drugs for “blood and blood-forming organs” exhibit high potential to be repositioned as drugs for “anti-parasitic products, insecticides, and repellents,” and vice versa. Moreover, “anti-infective drugs for systemic use” show higher potential to be repositioned to drugs for “respiratory system”.

We assessed the repositioning potential of drugs by using side effect data from the Side Effect Resource (SIDER) database to investigate the relationship between novel therapeutic properties based on transcriptome responses and properties based on the side effects of drugs. A total of 247 drugs were found in both the DTN-T and the SIDER database. We measured the similarity of these 247 drugs using the Jaccard coefficient, and we constructed the drug similarity network based on side effect data (Supplementary Table 2). According to the ATC code of drugs, we divided the 247 drugs into 14 communities, in which drugs with multiple ATC codes belonged to multiple communities. We computed the node-to-community coefficient for each drug, and constructed a drug therapeutic property network based on side effect data, denoted as DTN-SE (Supplementary Table 3). In the DTN-SE, a feature vector with 14 dimensions was denoted for each drug, thereby measuring the similarity of the drug and the 14 ATC communities. In the DTN-T, a feature vector with 14 dimensions was defined for each drug, thereby measuring the potential of the drug for repositioning to 14 ATC communities. We explored the relationship between the DTN-T and DTN-SE by computing the Spearman correlation coefficient across these two networks (Supplementary Table 4). The results demonstrated a positive correlation between the repositioning potential shown at the transcriptome level and at the side effect level for a majority of drugs (Fig. 3C). This finding suggested that a drug with high similarity with drugs in a certain ATC community based on side effect data shows high potential for repositioning to the ATC therapeutic property.

Similarly, we assessed the repositioning potential of drugs by using two-dimensional structure data from the PubChem Compound database to investigate the relationship between novel therapeutic properties based on transcriptome responses and properties based on the drug structures. We measured the structural similarity of the 247 drugs using the Tanimoto coefficient, constructed the drug similarity network based on structure data, and divided the drugs into 14 communities according to the ATC codes (Supplementary Table 5). We calculated the node-to-community coefficient for each drug and constructed a drug therapeutic property network based on structure data, denoted as DTN-ST (Supplementary Table 6). Moreover, we explored the relationship between DTN-T and DTN-ST by computing the Spearman correlation coefficient across these two networks (Supplementary Table 7). As above, a positive correlation exists between the repositioning potential at the transcriptome level and at the side effect level for most drugs (Fig. 3D). This finding suggested that a high similarity of a drug with drugs in a certain ATC community based on structure data denotes a high potential of the drug to be repositioned to the ATC therapeutic property.

### Case studies

In the DTN-T, we used the term “star drug” for any drug that was predicted to have more than 0.9 probability to be repositioned to at least one novel ATC therapeutic property except for known ATC labels. A total of 98 “star drugs” were identified (Supplementary Table 8). Notably, 5 “star drugs”, as denoted by SD1, were predicted to have probability = 1 to be repositioned to novel therapeutic properties. The correlation coefficient of 10 “star drugs” between their transcriptome responses and side effects exceeded 0.4, and the group was denoted by SD2. The correlation coefficient of 14 “star drugs”, denoted by SD3, between their transcriptome responses and structures exceeded 0.4. We combined SD1, SD2, and SD3 as a highly convincing “star drug” set (Supplementary Table 9). Two drugs, zonisamide and brinzolamide, were in both SD1 and SD2.

Zonisamide, a drug for “nervous system” indications that is used to treat epilepsy, was predicted to be repositioned to drugs for the “cardiovascular system” with 0.945 probability. A study in 1987 already found that zonisamide can lower blood pressure and decrease blood flow in the carotid and femoral arteries of anesthetized dogs^35^. Moreover, we investigated the targets of the drug and found 31 drug targets of zonisamide. The mutations of SCN1B, SCN2B, SCN3B, and SCN5A, which are the targets of zonisamide, can lead to Brugada syndrome^36^. The mutation of SCN3B, another target of zonisamide, may be associated with abnormal cardiac electrophysiology^37^. All of the above drug targets are encoded by sodium ion channel-related genes. Moreover, the target CACNA1G is related to cardiac pacing activity. Furthermore, the activation of target CA1 is closely related to human ischemic diabetic cardiomyopathy^38^. These results suggested that zonisamide could regulate cardiac electrophysiology activity by targeting sodium ion channel-related genes and ultimately exert a therapeutic effect on cardiovascular disease. We used the STITCH database to analyze the interacting protein of zonisamide and found 81 interacting proteins, thereby suggesting that zonisamide is potent to be repositioned to other therapeutic properties^39^.

Furthermore, we used the Fisher exact test to statistically examine the Human Phenotype Ontology enrichment of downregulated genes when zonisamide stimulated the PC3 cell line. We found that the downregulated genes were enriched in Ventricular tachycardia (HP:0004756) (Fisher exact test, p = 0.025), thereby providing another piece of evidence in favor of the prediction result. Among all drugs for the “cardiovascular system” in the DTN-SE, zonisamide was the most similar to doxazosin, a drug for treating mild or moderate hypertension and urinary obstruction. In the DTN-ST, zonisamide shared the largest similarity with bumetanide, a drug for treating congestive heart failure and nephrotic syndrome.

Brinzolamide, a drug for “sensory organs” that is used to lower intraocular pressure in patients with open-angle glaucoma or ocular hypertension, was predicted to be repositioned to treat the “cardiovascular system” with 0.935 probability. A total of 4 drug targets and 39 interacting proteins were found in brinzolamide^39^. CA1, a target of brinzolamide, is closely related to human ischemic diabetic cardiomyopathy^38^. Moreover, we used the Fisher exact test to statistically investigate the KEGG and GO enrichment of downregulated and upregulated genes when brinzolamide stimulated the PC3 cell line. The downregulated genes are enriched in a dilated cardiomyopathy pathway (hsa05414) (p = 0.048), cardiac myofibril assembly (GO:0055003) (Fisher exact test, p = 0.026), and blood coagulation (GO:0007597) (Fisher exact test, p = 0.031). The upregulated genes are enriched in regulation of blood pressure (GO:0008217) (Fisher exact test, p = 0.016). Among all drugs for “cardiovascular system” in the DTN-SE, brinzolamide was the most similar to hydrochlorothiazide, a drug for treating hypertension and edema. In the DTN-ST, brinzolamide was the most similar to nifedipine, a drug used to manage angina, high blood pressure, Raynaud’s phenomenon, and premature labor.

## Discussion and Conclusions

In this research, we used a machine learning algorithm for multi-label classification to assess 480 approved drugs for their novel therapeutic properties and repositioning potentials based on transcriptome data under drug perturbations from the LINCS L1000 database. We found that drugs with different therapeutic properties exhibit different repositioning potentials. For instance, drugs for “blood and blood forming organs” show high potential to be repositioned to drugs for “anti-parasitic products, insecticides, and repellents”, whereas drugs for “anti-infective for systemic use” tended to be repositioned to drugs for “respiratory system”.

Therefore, we investigated whether the frequency of a novel therapeutic property is related to the number of drug targets. We found that the frequency of a novel therapeutic property is not significantly correlated with the drug target number (Spearman correlation coefficient = 0.037). Moreover, we considered the drug/chemical–protein interaction information of the STITCH database and found that the novel therapeutic property is not significantly correlated with the number of interacting proteins (Spearman correlation coefficient = −0.029), which may be ascribed to the contingency of drug target discovery and the incompleteness of the drug–target map^39^. Drugs with high repositioning potential but few targets may be due to the drugs being newly approved or other factors. This finding inspires us to predict and discover more potential therapeutic targets in the future using machine learning.

Furthermore, we deciphered the relationships among transcriptional responses, side effects, and the structure of drugs. If a drug is similar to a certain ATC community based on side effect data, considering all the drugs with the ATC therapeutic property, the drug may have high potential to be repositioned to the therapeutic property. The same is true for structure data. This finding encourages us to develop a more precise drug repositioning strategy by integrating the transcriptional responses, side effects, and structure properties of drugs.

To this end, we discussed two drugs, zonisamide and brinzolamide, which have a high correlation coefficient between transcriptional responses and side effects, as well as between transcriptome responses and structure. These drugs were originally intended for the treatment of epilepsy and glaucoma, respectively, but both were predicted to be repositioned to the therapeutic property for “cardiovascular system”. Moreover, the potential of zonisamide for lowering blood pressure has previously been reported. Regarding drug targets and cellular responses, we discovered that zonisamide and brinzolamide are closely related to cardiovascular biochemical pathways and relevant biological processes.

Moreover, five drugs were predicted to be repositioned to novel therapeutic properties with probability = 1. For example, candesartan was originally intended to treat hypertension and myocardial infarction but was predicted to be repositioned to drugs for the “musculoskeletal system”. A study by Hong K *et al*. illustrated that as an inhibitor of AT1R, candesartan can partially suppress myogenic responses^40^. The activity of candesartan to block AT1R acutely could lead to the recruitment of the microvascular system in skeletal and cardiac muscles^41^.

One limitation is that the LINCS drug perturbation datasets are limitedly released. For the completeness of this work, we only selected the most completed drug perturbation datasets in the PC3 cell lines to train the Softmax model and achieve drug repositioning. Moreover, we applied our method to LINCS drug profiles in other cell lines. Few approved drugs were in these cell lines; however, our prediction method still exhibited high training and validation accuracy (Supplementary Materials). These discoveries proved that the machine learning algorithm, Softmax, has high potential and wide prospects for predicting drug repositioning from another aspect.

## Materials and Methods

In this section, we discuss how we accomplished drug repositioning by using machine learning algorithms, including data preprocessing and problem modeling. Moreover, we present the machine learning algorithms used for training and prediction.

To explore new therapeutic properties of drugs, we approached the problem as a multi-label classification task in the machine learning domain. As input datasets, we obtained L1000 transcriptional profiles when the PC3 cell line was exposed to 480 FDA-approved drugs. We divided the input datasets into two groups: training and validation sets. In the profile data from the L1000 database, drug perturbation trials were regarded as samples, which were labeled according to 14 ATC codes of drugs from the DrugBank database, and 978 landmark genes were regarded as features. We trained our model through machine learning algorithms based on the profile data and predicted new labels for samples in the validation sets.

### Drug perturbation data from the L1000 database

The LINCS project, launched in 2010, has created a network-based understanding of multilevel cellular changes when cells are exposed to various perturbing agents. LINCS hopes to decipher how cells respond to various genetic and environmental stressors. The pilot phase of the project was completed in 2013 and generated more than 660,000 gene expression profiles, among which more than 6,000 small molecule compounds stimulated 72 cell lines in various doses.

The LINCS L1000 biotechnology is a new technology that measures the expression of only 978 landmark genes using the correlation of genes to infer the remaining ~20,000 gene expressions. The data structure of this project, similar to the TCGA project (https://cancergenome.nih.gov/), has four levels. Level 1 data refers to the expression value of the 978 landmark genes, and Level 2 data refers to the normalized expression value of the 978 landmark genes. Level 3 data records genome-wide gene expression, whereas Level 4 data records the Z-score of genome-wide gene expression.

In this paper, we used Level 4 data of drug perturbations in the PC3 cell line.

### DrugBank database

DrugBank, a comprehensive drug data resource, records the chemical, pharmacological, and pharmaceutical features of more than 8,000 drugs, including 2016 FDA-approved drugs^9^. We used version 5.0 of the DrugBank database in this paper. To make the cross-platform comparisons compatible, we considered the PubChem ID as the identifier of drugs across the DrugBank and LINCS databases. In the machine learning process, we used the first level of ATC classification codes, which indicate drug therapeutic properties, to label drugs.

### Data preprocessing

All the perturbation trial numbers in the LINCS project to the approved drugs in the DrugBank database are difficult to map because the LINCS project is still in progress. We selected the Level 4 data of 480 FDA-approved drug perturbations in the PC3 cell line, which included 4823 samples. To reduce the feature dimension, we used the Z-score of only 978 landmark genes that have been proven to be able to represent genome-wide expressions effectively.

For all trials of a given drug, we calculated the Pearson correlation coefficient matrix. Then, we employed the *k*-means method to divide the trials into several groups and selected the group with the maximum intra-class Pearson correlation coefficient as the representation of the drug, denoted by S1. To retain more information on these drug trials, we averaged the data from all trials as an independent sample, S2. Last, we established a credible set, S, of this drug by combining S1 and S2.

We used the first level of ATC codes as the labels of the drugs and obtained 14 labels in total. For a drug with multiple labels, we retained all labels for multi-label classification.

### Problem Definition

In this work, the input data were obtained from L1000 and the DrugBank. Drug perturbation trials from the L1000 database were regarded as samples. The samples were labeled by ATC codes from the DrugBank database, and 978 landmark genes were regarded as features. This approach implies that drugs with multiple ATC categories may exhibit multiple therapeutic properties. Our intention was to predict new ATC categories of drugs; thus, for our work, drug repositioning was modeled as a multi-label classification task, which is the problem of categorizing instances into more than or equal to one class. The input space consisted of all drug perturbation samples with labels. Both data and labels were discrete random variables. All labels were defined by binary variables, which generated a multi-dimensional vector. The details of the definitions are as follows:

**Definition 6**: Drug matrix *DM* is an *m* by *n* matrix that contains all data samples of the drug set. *m* is the number of samples, and *n* is the number of features. Each line represents one sample.

**Definition 7**: Feature *DM*_*i*,*j*_ is a real number that corresponds to the expression of the *j* ^th^ gene for sample *i*.

**Definition 8**: Label matrix *LB* is an *m* by *q* matrix. *LB*_*i,j*_ is one label for drug *i*. If *LB*_*i,j*_ is 1, then drug *i* has an effect on disease *j*; otherwise, drug *i* does not have an effect on disease *j*. *LB*_*i*_ is the label vector of drug *i*.

**Definition 9**: Classification matrix *CF* is an *m* by *q* binary matrix. Each line represents categories to which one sample belongs. If *CF*_*i*,*j*_ is 1, then sample *i* belongs to category *j*, and a set of classified categories can exist for each sample. Otherwise, the set does not exist.

The drug matrix *DM* and label matrix *LB* were fed into the classifier for training and validation; then, the classification matrix *CF* was regarded as output space.

### Supervised Learning

Hypothesis space *F* is the set of all conditional probability distributions or decision functions, shown as Eq. 1. Assuming that the decision function is a linear function of the input variable, the hypothesis space is a set of all linear functions:

1 $$F=\{\,f\,|{Y}^{\ast }=f(X)\}$$

In supervised learning, given an infinite number of models in the hypothesis space, if model *f* is selected as a decision function, a predicted value Y* = *f*(*X*) will result for any input *X*. The objective function, a non-negative real-valued function of *f*(*X*) and *Y*, is constructed for evaluating the accuracy of training and defined by *L*(*Y*, *f*(*X*)), where *L* measures whether the predicted value is or is not close to the true value. As the loss value of the object function decreases, the model fits the training sets better. If the inputs and outputs (*X*, Y*) of the model follow a joint distribution P(*X*, Y*), the model with expected loss minimization, defined as Eq. 2, is selected by the machine learning algorithm:

2 $${R}_{exp}(f)=\,{E}_{p}[L(Y,\,{Y}^{\ast })]\,=\,{f}_{x\times y}L(Y,\,{Y}^{\ast })P(x,\,y)dxdy$$

### SVM, Random Forest, and CNN

For binary classification tasks in the machine learning domain, the conventional logistic regression model and classical SVM have shown amazing performance in practical problems^42^. As a discriminative model, SVM models conditional probability distribution P(*Y*|*X*) directly through learning on training sets. SVM builds a high-dimensional (even infinite dimensional) hyperplane, which is called the decision boundary, for classifying samples. The support vectors refer to the sample points that are closest to the decision boundary, with a larger margin, indicating a better fit of SVM to the training sets; thus, SVM is called a “large margin classifier”. However, multi-label classification tasks are challenging for SVM because expensive computation is required if an objective function is rebuilt with respect to all parameters of all classes. Otherwise, the machine learning algorithm will suffer from class imbalance when a multi-label SVM is established as one-versus-all.

Several supervised learning algorithms are suited to classify instances into a multiclass space. Random forest is a classifier that combines a forest of decision trees grown on random input vectors and splits nodes on a random subset of features according to information gain rate; the random forest is recognized as a robust classifier^43^ because of eliminating the disadvantage of instability for the decision trees and showing the capacity to cope with large feature spaces. In fact, feature selection is implicitly incorporated during each tree construction. At each node of one of the decision trees in the forest, the best variable to split on a random subset of variables is selected. During classification, only the features needed for the test pattern under consideration are involved^44^. However, the incomplete value of features in practical problems affects the best performance of RF.

Convolutional neural network (CNNs) have been used widely in various vision tasks, such as image classification^45^, object detection^46^, and object tracking^47^ because of their capacity to filter data noise and extract high-level abstract features. The feature extractor in CNN is stacked by a convolution layer and a max pooling layer iteratively, followed by a fully connected layer for combining high-level features. The combined features in the last layer of a CNN are a valid replacement representation of the original data and improve the performance of a classifier. Finally, the classification layer is charged by a Softmax classifier for multi-label classification. However, a CNN can be overfitted owing to its complex structure.

### Softmax Regression Model

The logistic regression model, whose common hypothesis and objective function are defined by Eqs 3 and 4, looks for decision boundaries by optimizing the objective function. Not only does this kind of sigmoid function transform input to non-linear form, the function also normalizes the output into a specific range.

3 $${h}_{\theta }(x)=\frac{1}{1+\exp (-{\theta }^{T}x)}$$

4 $$J(\theta )=-\frac{1}{m}[\sum _{i=1}^{m}{y}^{(i)}\,\mathrm{log}\,{h}_{\theta }({x}^{(i)})+(1-{y}^{(i)})\mathrm{log}(1-{h}_{\theta }({x}^{(i)}))]$$

The Softmax regression model^48^ is an extension of the logistic regression model to handle classification problems, in which the true value is a *q*-dimensional binary vector (3 ≤ *q*) *LB*_*i*_ (1 ≤ *i* ≤ *m*). In this work, a fully connected network with the same architecture as two layers of perceptron was designed with Softmax as the activation function to implement the Softmax regression model. The number of neurons in the input layer corresponded to the number of feature *n* in each sample, and the number of neurons in the classification layer equals to the number of classes *q*. For a given input *x*, the model will give the probabilities P(*y* = *j* | *x*) for each class *j*. Therefore, each *x* corresponds to *LB*_*i*_ (1 ≤ *i* *≤* *m*), and the sum of all elements of *LB*_*i*_ is 1. The hypothesis of the Softmax regression model is defined as Eq. 5:

5 $${h}_{\theta }({x}^{(i)})=[\begin{array}{c}P({y}^{(i)}=1|{x}^{(i)};{\theta }_{1})\\ P({y}^{(i)}=2|{x}^{(i)};{\theta }_{2})\\ \ldots \\ P({y}^{(i)}=q|{x}^{(i)};{\theta }_{q})\end{array}]=\frac{1}{{\sum }_{j=1}^{q}{e}^{{\theta }_{j}^{T}{x}^{(i)}}}[\begin{array}{c}{e}^{{\theta }_{1}^{T}{x}^{(i)}}\\ {e}^{{\theta }_{2}^{T}{x}^{(i)}}\\ \ldots \\ {e}^{{\theta }_{q}^{T}{x}^{(i)}}\end{array}]$$

*θ*_*1*_, *θ*_*2*_, …, *θ*_*q*_ are *n*-dimensional parameter vectors. For convenience and vectorization, *θ*_*i*_(1 ≤ *i* ≤ *q*) is inserted one by one into a *q* by (*n* + 1) parameter matrix, including the bias unit. For multi-label classification tasks, the adoption of Softmax regression or *q* binary classifiers depends on whether the classes are mutually exclusive.

### Model Training

Model training is a process for optimizing the objective function by using a learning algorithm. However, several models with various structural complexities exist in the hypothesis space. If a “true” model exists in the hypothesis space, the model we selected is supposed to approximate the said model. Specifically, the number of parameters or parameter vectors should be similar between the “true” model and model we choose.

Here, the drug repositioning problem was transformed from a multi-label classification task to the optimization of the objective function, and the learning algorithm was responsible for looking for the optimal solution. However, in many practical problems, analytic solutions do not exist; therefore, so in this work, we adopted a mini-batch gradient descent for optimization.

The cross entropy function as the objective function is defined by Eq. 6. Compared with the mean square error function, the cross entropy function effectively solves the problem wherein that the convergence time is too long when the gradient is too small.

6 $$L(Y,f(X))=-\frac{1}{n}\sum _{x}\,\sum _{y}y\times \,\mathrm{ln}(f(x))$$

Once training accuracy (as defined in Eq. 7) converges, the training process is complete; moreover, the most significant factor for our model was its generalization ability. Not only does a machine learning algorithm train a model to fit training sets, but the model also must generalize new data. To avoid over-fitting, the regularization term should be added into the objective function to minimize structural risks. Last, training and validation errors were used to evaluate how well the model fits to training sets and to assess its generalization ability.

In this work, the whole drug data was separated into K validation sets to evaluate generalization ability. On training folds, its mean loss (empirical risk) is defined by Eq. 8. According to the law of large numbers (LLN), when the number of samples tends to be unlimited, the empirical risk obtained from a large number of trials should be close to the expected loss.

7 $$Training\_accuracy=\frac{|\{i|cp(i),i\in Training\_\,fold\}|}{|Training\_\,fold|}$$

8 $${R}_{emp}(f)=\frac{1}{m}\sum _{i=1}^{m}L({y}_{i},f({x}_{i}))$$

Moreover, the structural risk item will be added into the objective function (as defined in Eq. 9). Therefore, the learning targets are empirical risk minimization and structural risk minimization. The former can guarantee that the model will fit to training sets well, and the latter is equivalent to the regularization that can avoid over-fitting effectively. *J*(*f*) represents the complexity of the model and *λ* will keep the tradeoff between empirical and structural risks. The model with low structural risk will have high generalization ability on the new data. The performance of the trained model was assessed by validation accuracy (as defined in Eq. 10) and such criterion measured the generalization ability of the model.

9 $${R}_{srm}(f)=\frac{1}{m}\sum _{i=1}^{m}L({y}_{i},f({x}_{i}))+\lambda J(f)$$

10 $$Validation\_accuracy=\frac{|\{i|cp(i),i\in Validation\_\,fold\}|}{|Validation\_\,fold|}$$

### Enrichment Ratio of therapeutic property

We calculated the ER to measure the repositioning potential between drug therapeutic properties. The ER of drugs with X label repositioning to those with Y label is defined as Eq. 11:

11 $$E{R}_{X,Y}=(\frac{a}{b})/(\frac{c}{d})$$

where *a* is the number of drugs with X label repositioning to Y label, *b* is the number of drugs with any labels repositioning to Y label, *c* is the number of drugs with X label repositioning to any labels, and *d* is the number of drugs with any labels repositioning to any other labels.

### Drug similarity network based on side effect data

The SIDER is a database that records large amounts of information on drug side effects as obtained from published research by text mining^19^. For this paper, we used SIDER 4.1 to construct a drug similarity network based on side effects. The number of drugs found to overlap across SIDER, PubChem Compound, and LINCS was 247 in total.

We used all of the drug side effect relation pairs in the SIDER database to construct the drug–side effect network, an undirected and unweighted graph. Taking side effect occurrence as a feature of drugs, we measured the similarity of drugs by using the Jaccard coefficient, as shown in Eq. 12, where the Jaccard coefficient ranges from 0–1. If A and B are empty sets, the Jaccard coefficient between A and B is 1. Therefore, we constructed a similarity network based on side effects involving 247 FDA-approved drugs.

12 $${\rm{J}}({\rm{A}},{\rm{B}})=\frac{|{\rm{A}}{\cap }^{}{\rm{B}}|}{|{\rm{A}}{\cup }^{}{\rm{B}}|}=\frac{|{\rm{A}}{\cap }^{}{\rm{B}}|}{|{\rm{A}}|+|{\rm{B}}|-|{\rm{A}}{\cap }^{}{\rm{B}}|}$$

### Drug similarity network based on structure data

PubChem Compound is a database that records the chemical description data of various drugs^49^. For this paper, we used the two-dimensional structure data of drugs to construct a drug similarity network based on structure.

We quantified the two-dimensional structures of drug molecules by using atom pair descriptors and measured the similarity of drugs using the Tanimoto coefficient, as shown in Eq. 13:

13 $$T(A,B)=c/(a+b-c)$$

where *a* is the number of atom pairs in drug A but not drug B, *b* is the number of atom pairs in drug B but not drug A, and *c* is the number of atom pairs in both drugs A and B.

Therefore, we constructed a similarity network based on the two-dimensional structure involving 247 FDA-approved drugs.

## Electronic supplementary material

Supplementary Table 1

Supplementary Table 2

Supplementary Table 3

Supplementary Table 4

Supplementary Table 5

Supplementary Table 6

Supplementary Table 7

Supplementary Table 8

Supplementary Table 9

Supplementary Materials
