# Supplementary material for: Discovery of novel therapeutic properties of drugs from transcriptional responses based on multi-label classification
Source: Sci Rep. 2017 Aug 2;7:7136. doi: 10.1038/s41598-017-07705-8 (PMC5541064; doi:10.1038/s41598-017-07705-8)
Supplement: Supplementary file 10 — Supplementary Materials [file 41598_2017_7705_MOESM10_ESM.doc]

# Discovery of novel therapeutic properties of drugs from transcriptional responses based on multi-label classification

Lingwei Xie ^1,+^, Song He ^2,+^, Yuqi Wen ^2^, Xiaochen Bo ^2^, Zhongnan Zhang ^1,*^

^1^ Software School, Xiamen University, Xiamen Fujian 361005, P.R. China

^2^ Beijing Institute of Radiation Medicine, Beijing 100850, P.R. China

*Correspondence to [zhongnan_zhang@xmu.edu.cn].

^+^These authors contributed equally to this work and should be considered as co-first authors.

Prediction result in other cell lines

We use the prediction paradigm to predict the therapeutic properties of drugs in other cell lines. As illustrated in Supplementary Figure 1, the training accuracy in each cell line was about 80%, and the validation accuracy in each cell line was approximately 70%, which is similar to the accuracy in the PC3 cell line. Then we compare the new therapeutic properties of drugs across six main cell lines, and we find that the prediction result is highly correlated across cell lines as the following Supplementary Table 1 and Supplementary Table 2. This evidence supports that our prediction model is correct and the result of drug repositioning is stable across cell lines.

Supplementary Table 1. Spearman correlation coefficient across the prediction of six main cell lines in the LINCS project.

|  | PC3 | A549 | HA1E | MCF7 | NPC | VCAP |
| --- | --- | --- | --- | --- | --- | --- |
| PC3 |  | 0.6168 | 0.5216 | 0.5730 | 0.4927 | 0.5834 |
| A549 |  |  | 0.5810 | 0.6048 | 0.5036 | 0.6333 |
| HA1E |  |  |  | 0.4391 | 0.4442 | 0.5035 |
| MCF7 |  |  |  |  | 0.5204 | 0.6045 |
| NPC |  |  |  |  |  | 0.4824 |
| VCAP |  |  |  |  |  |  |

Supplementary Table 2. P-value of correlation coefficient across the prediction of six main cell lines in the LINCS project.

|  | PC3 | A549 | HA1E | MCF7 | NPC | VCAP |
| --- | --- | --- | --- | --- | --- | --- |
| PC3 |  | 7.72*10^-104^ | 1.64*10^-51^ | 1.37*10^-163^ | 4.45*10^-47^ | 1.41*10^-155^ |
| A549 |  |  | 5.25*10^-23^ | 4.47*10^-25^ | 2.90*10^-9^ | 1.32*10^-42^ |
| HA1E |  |  |  | 5.44*10^-13^ | 7.98*10^-8^ | 1.05*10^-18^ |
| MCF7 |  |  |  |  | 9.59*10^-20^ | 1.14*10^-20^ |
| NPC |  |  |  |  |  | 4.44*10^-20^ |
| VCAP |  |  |  |  |  |  |

| [A] | 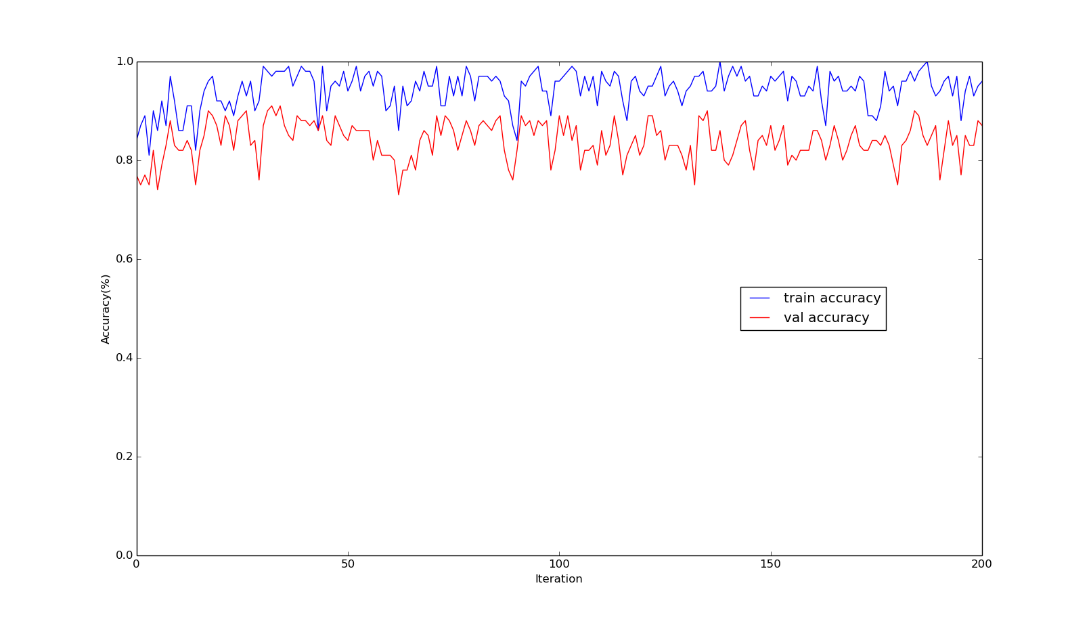 |
| --- | --- |
| [B] | 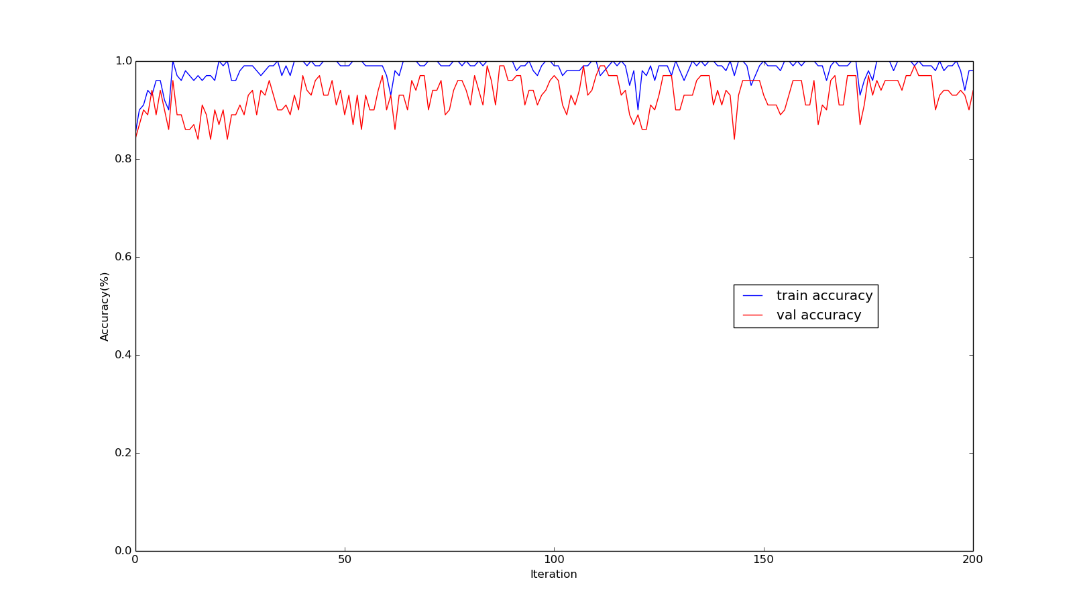 |
| [C] | 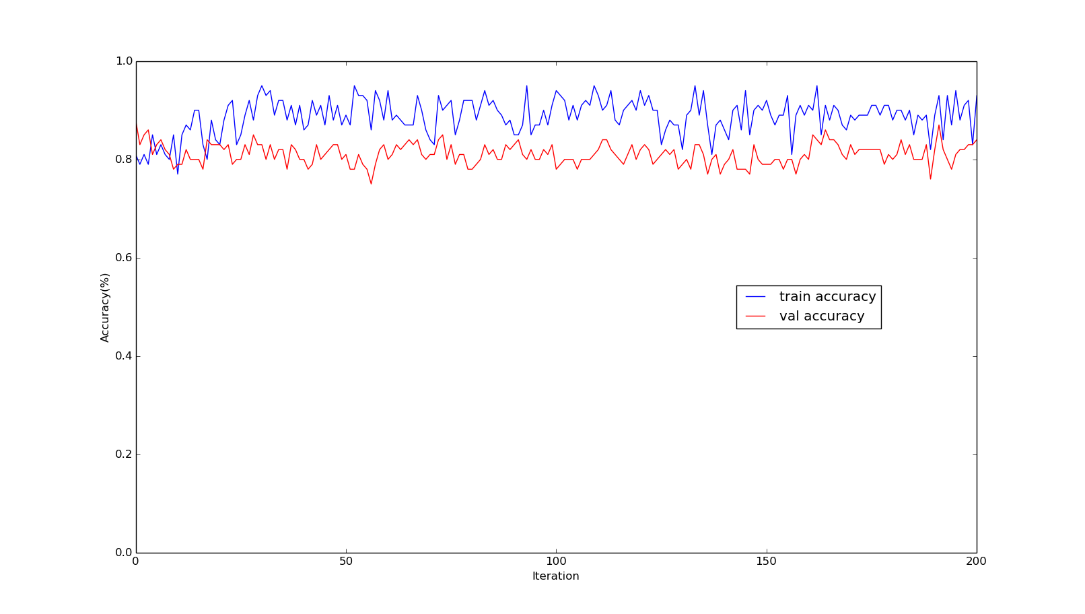 |
| [D] | 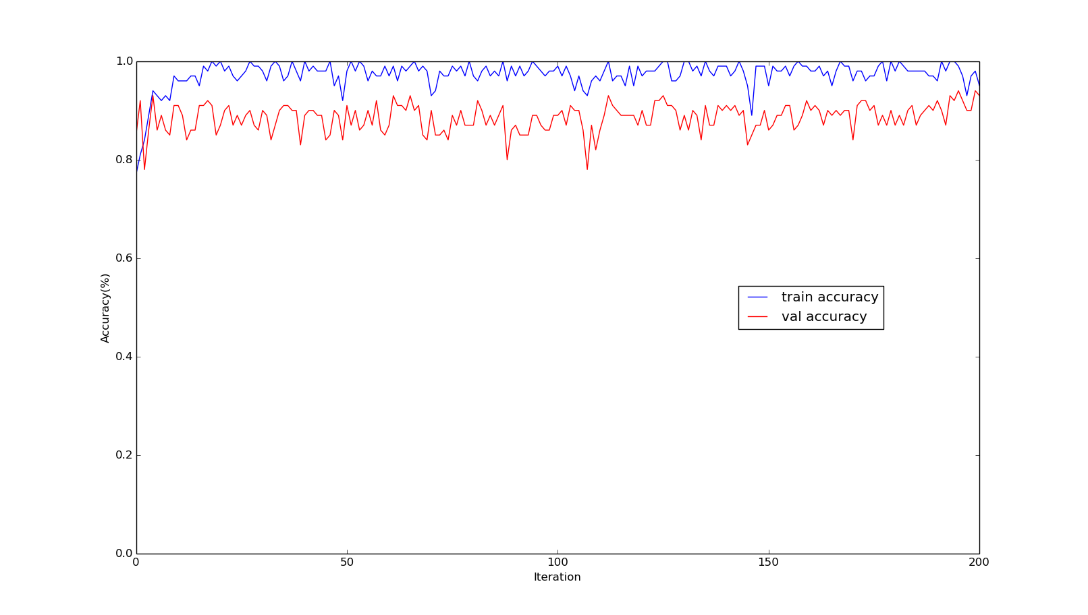 |
| [E] | 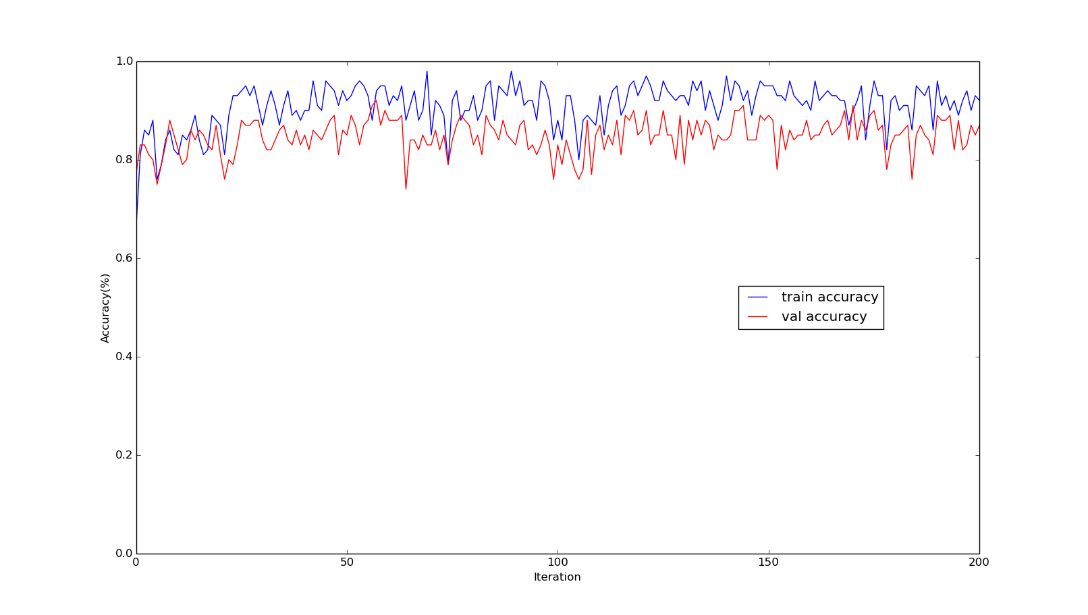 |

Supplementary Figure 1. The softmax result across other five main cell lines. [A] A549 cell line. [B] HA1E cell line. [C] MCF7 cell line. [D] NPC cell line. [E] VCAP cell line.
